# Gender-Specific Medicine in the European Society of Cardiology Guidelines from 2018 to 2023: Where Are We Going?

**DOI:** 10.3390/jcm13144026

**Published:** 2024-07-10

**Authors:** Federica Piani, Laura Baffoni, Enrico Strocchi, Claudio Borghi

**Affiliations:** 1 Hypertension and Cardiovascular Risk Research Center, Medical and Surgical Sciences Department, Alma Mater Studiorum University of Bologna, 40138 Bologna, Italy; enrico.strocchi@unibo.it (E.S.); claudio.borghi@unibo.it (C.B.); 2 Center for Gender Medicine OMCEO Rimini, 47923 Rimini, Italy; laura.baffoni@omceo.rn.it

**Keywords:** gender medicine, gender equality, sex differences, cardiovascular guidelines, European Society of Cardiology

## Abstract

**Background/Objectives**: Evidence-based medicine (EBM) shapes most clinical guidelines. Although the advent of EBM marked a significant advancement, failure to include sex differences in the study design and analysis of most trials leads to an under-representation of gender-specific medicine (GM) in EBM-directed guidelines. In this review, we evaluated how the topic of GM was developed in the guidelines produced by the European Society of Cardiology (ESC) from 2018 to 2023. **Methods**: Two independent reviewers evaluated 24 ESC guidelines. Significant mentions of GM were counted and divided between epidemiology, diagnosis, and therapeutics. The qualitative and semi-quantitative analysis of information relating to GM was performed. Data on the number of citations of papers with a title concerning GM and the prevalence and role of women in guidelines’ authorship were also analyzed. **Results**: Less than 50% of guidelines had a section dedicated to GM. Only nine guidelines were led by a woman, and 144/567 authors were female. In the most recent guidelines and in those with at least 30% of female authors, there was an increased mention of GM. On average, guidelines had four significant mentions of GM regarding epidemiology, two regarding diagnosis, and one regarding therapy. Articles with titles concerning GM made up, on average, 1.5% of the total number of citations. **Conclusions**: Although sex differences play a significant role in most clinical scenarios, ESC guidelines still do not sufficiently account for this. The problem does not seem to solely lie in the guidelines, but in the lack of attention to GM in research needed for their preparation.

## 1. Introduction

Since the 1990s, evidence-based medicine (EBM), i.e., “the conscientious, explicit and judicious use of the best current evidence in making decisions regarding individual patients”, has gained traction [1]. This means integrating individual clinical expertise with the best external clinical evidence provided by systematic research. EBM constitutes a new approach to healthcare where “clinical decisions result from the integration between the doctor’s experience and the use of the best scientific evidence available, relating to the accuracy of diagnostic tests, the effectiveness/safety of preventive, therapeutic and rehabilitative treatments”. The latter are summarized in the guidelines produced by scientific societies through a process that reflects the rules of EBM, representing an indispensable resource for a doctor’s daily activity [2].

Around the same period, gender medicine was also developing. Indeed, in 1994, the US National Institutes of Health (NIH) issued a guideline to study and evaluate gender differences in clinical trials to ensure that the safety and efficacy of drugs would be thoroughly investigated across the full spectrum of patients who would use the therapy [3]. Gender-specific medicine (GM) refers to the study of how biological differences (sex) and socio-cultural differences (gender) influence physiology and pathology and therefore health status and disease manifestations, epidemiology, diagnosis, response to treatments, and prevention strategies in all individuals. It aims to personalize healthcare to improve the effectiveness and equity of treatments for everyone. Understanding the impact of sex and gender on biology and medicine is crucial in clinical research, and this should be reflected in clinical guidelines [4,5]. In particular, gender differences have been known in the field of cardiovascular diseases since Healy’s article [6]. The European Society of Cardiology (ESC) is committed to reducing the burden of cardiovascular disease and enhancing the standards of care for all patients with cardiovascular conditions. Between 2020 and 2022, the ESC has developed a Gender Policy to be congruent with the general positions of the European Institute for Gender Equality and enhance female and GM representation in all ESC publications, including scientific statements and guidelines. Gender Policy is of crucial importance considering that ESC guidelines have a significantly lower number of female authors compared to American College of Cardiology/American Heart Association (ACC/AHA) and Canadian Cardiovascular Society (CCS) guidelines [7]. Female authors accounted for less than 14% of the total number of authors in ESC guidelines from 2006 to 2015 compared to 30% for CCS and 20% for ACC/AHA guidelines.

The aim of the present study was to evaluate how the topic of GM was developed in the guidelines produced by the ESC and to assess gender differences in authorship and leading positions.

## 2. Materials and Methods

Two independent reviewers evaluated ESC guidelines from 2018 to 2023 [8,9,10,11,12,13,14,15,16,17,18,19,20,21,22,23,24,25,26,27,28,29,30,31]. The timeframe of the guidelines’ selection was chosen based on the years of ESC Gender Policy development (2020–2022). Since guidelines released during the ESC Gender Policy are those from 2021 to 2023 (3 years span), the authors decided to also include guidelines up to three years before the ESC Gender Policy (from 2018 to 2020). Guidelines were extracted from the guidelines section of the ESC website.

The table of contents of each guideline was checked to see whether a chapter or a section or a sub-section was dedicated to sex or gender differences (the two terms are often used interchangeably in the guidelines); the sections “key-points” and “gaps in evidence” were also searched for any mention of sex differences; then, the text of each guideline was analyzed to identify any information containing male–female differences regarding epidemiology, diagnosis, or therapy. At first, an automatic search of the words “gender”, “sex”, “male”, “female”, “men”, and “women” was performed; then, the content of each sentence containing any of these key words was evaluated and classified as relevant or not for sex or gender differences in epidemiology (incidence or age at onset of a disease, access to diagnostic or surgical intervention, etc.…), diagnosis (risk scales, diagnostic cut-off for laboratory values, preferred strategies, etc.), or treatment (preferred drugs, side effects, best time of surgical intervention, etc.…). With a similar strategy, the bibliography of each guideline was also examined, and all the articles with a title containing at least one of the key words mentioned before were analyzed. Guidelines on the management of cardiovascular disease during pregnancy were not considered in the analysis, except for the assessment of female authorship. The authorship of each guideline was assessed for the total number of authors and number of female authors, with attention paid to the first and last authorship position.

### Statistical Analysis

As for descriptive statistics, the total number of significant mentions of sex differences for each area (epidemiology, diagnosis, therapeutics) and the total number of citations of articles with a title concerning sex differences were expressed as the mean and standard deviation (SD) or the median and interquartile range (IQR) according to variable distribution. The presence of at least one section or sub-section on GM, the prevalence of female authors being at least 30% of the total number of guidelines’ authors, and the position of the first or last author covered by a woman were expressed as dichotomous variables. Differences in the presence of GM mentions in guidelines led by women or with at least 30% of female authors vs. those not led by women or with less than 30% of female authors were assessed with the Mann–Whitney U test or Chi-squared test according to the variable type and distribution. The cut-off of 30% was chosen based on the 60th percentile of percentages of female authors. Two-tailed *p*-values < 0.05 were considered statistically significant. The analyses were conducted using the Statistical Package for the Social Science (SPSS) version 28 (SPSS Inc., Chicago, IL, USA), Macintosh version.

## 3. Results

Since 2018, the ESC has produced 24 guidelines that are listed in Table 1 along with the year of publication, the total number of pages, and the total number of references.

The cited articles are generally very numerous, suggesting the tremendous effort spent in drafting the guidelines. The number assigned to each guideline in Table 1 is also used to identify the same guideline in the following tables and where they are mentioned in the subsequent analyses. Table 2 summarizes the number of female authors, the first and last authorship position covered by women, the presence of a chapter or a section dedicated to sex differences or to pregnancy, and the presence of sentences on sex differences in the sections “key points” and “gaps in evidence”.

A chapter or a section dedicated to sex differences was present in only 11 of 23 guidelines, with a trend towards an improvement in most recent guidelines, although in many cases, this was limited to short sections or sub-sections. Regarding pregnancy, the presence of specific sections on the topic was found in 16 of the 23 guidelines. Sex differences were cited in the “key points” section in only two guidelines, while the lack of sufficient information about sex differences was reported in 6 of 23 guidelines in the “gaps in knowledge” section. Sentences on GM in “key points” and “gap of knowledge” tended to be more common in most recent guidelines. Only 144 of 567 authors of the guidelines were female, and 9 guidelines out of 24 were led by women. No guideline from 2018 or 2019 was led by a woman. All 2018 guidelines had less than 30% female authors and all 2023 guidelines had more than 30% women among authors, suggesting that action has been taken to improve this gender equality parameter.

Table 3 lists the number of times sex differences key words were found in the text of each guideline. References to sex differences have been divided into epidemiology, diagnosis, or treatment.

In the last column of Table 3, the number of cited papers (and percentage of the total) with a title containing the abovementioned key words is reported. On average, these represented 1.5% of all citations. Mentions of GM are mostly found in the field of epidemiology, with only a few references to sex differences present in the areas of diagnosis and treatment, with two mentions of the topic on average.

No statistically significant differences in the number of mentions of GM in each area (epidemiology (*p* = 0.506), diagnosis (*p* = 0.825), therapy (*p* = 0.325)), nor in the number of cited works with a title concerning sex differences (*p* = 0.357), were observed between guidelines led by women compared to those led by men. When female authors made up more than 30% of the total number of authors of each guideline, we observed a significant increase in the number of cited papers with titles concerning sex differences (*p* = 0.019, mean rank 16.1 vs. 9.4). However, all other variables did not statistically differ between groups. Even if the differences did not reach statistical significance, guidelines led by women or with more than 30% female authors more frequently tended to have a section or sub-section on sex differences (5 out of 8 vs. 6 out of 15 guidelines and 6 out of 9 vs. 5 out of 14 guidelines in the groups of guidelines led by women and with good female representation, respectively).

## 4. Discussion

Clinical guidelines certainly represent a good summary of the available evidence and a point of reference for clinical practice [2]. While each physician can still make individual choices for each patient, these must be compared with what is reported in the guidelines on the basis of evidence. Prominent scientists have contributed to the latest ESC guidelines applying accurate EBM methodology. However, issues relating to sex differences are still poorly represented in the guidelines. Clinicians may encounter sex and gender disparities in epidemiology, pathophysiology, clinical manifestations, disease progression, and treatment outcomes. The main reasons for these differences are genetic or epigenetic or involve sex hormones and their receptors [32]. Fundamental sex differences, stemming directly from genetic heterogeneity between the X and Y chromosomes and parent-of-origin inheritance, exist at the molecular level in all human cells. A deeper understanding of sex differences and their underlying mechanisms is needed, as it holds the potential to develop new drugs that target sex-specific cardiovascular mechanisms and phenotypes. Comparing both sexes may reveal protective or maladaptive mechanisms unique to one sex, which could serve as novel therapeutic targets for one or both sexes.

In the present review, we found scarce representation of sex differences in the guidelines produced by the ESC. Cardiovascular diseases have significant sex-specific aspects involving incidence, diagnosis, prognosis, and therapy. For this reason, cardiovascular medicine has been widely studied in GM, being one of the first diseases evaluated by reviews on the topic [4]. The need to evaluate clinical guidelines regarding the presence of recommendations and evidence on sex and gender differences is reported in various papers. Naghipour theorizes the method of verifying the content of each guideline produced in the European Union in various fields of internal medicine using a specific research protocol that has not been used in any published research so far [33]. In the literature, there are examples of this type of research with less ambitious targets or simpler approaches. Griffin proposed an evaluation of four different hypertension guidelines based on the word count dedicated to sex- and gender-based medicine content [34]; this resulted in significant differences between the four guidelines examined, but, in agreement with our results, the authors found that the most mentioned sex-related topic was pregnancy. Tannenbaum searched clinical practice guidelines produced in Canada over a period of several years using the words “sex” and “gender” to identify useful insights into sex differences in diagnosis and treatment and concluded that these are present only in a minority of the guidelines taken into consideration [35]. We used a similar methodology limited to the guidelines produced by the ESC over a 6-year period; the 24 guidelines retrieved concern numerous areas of cardiovascular pathology and, having not carried out any selection, they constitute the entire production of guidelines of the scientific society. The exclusion of the guideline regarding pregnancy is justified by the purposes of the research. Attention to sex and gender differences has increased in recent years; however (i.e., in the latest guidelines on acute ischemic disease), most relevant information is in the appendix and not in the main text [36]. In general, the results of this work highlight that limited space is dedicated to sex- and gender-related differences in cardiovascular diseases. It must also be noted that in the guidelines examined, with the sole exception of that relating to cardiovascular prevention which adopts the definition of sex and gender proposed by the WHO, the two terms are used interchangeably and therefore inappropriately. This appears in line with the results of the massive work by Gogovor and colleagues on the integration of sex, as a biological attribute, and gender, as a socially constructed identity, in published reporting guidelines [37]. The authors found that among 407 reporting guidelines from 1995 to 2018, only one met their criteria (nonbinary, appropriate categorization, and non-interchangeability) for the correct use of sex and gender terms.

Even with the limits of a qualitative and semi-quantitative classification of useful information relating to sex and gender differences, this is clearly insufficient and concentrates on epidemiology above all. Since GM is not the medicine of women but an advancement in personalized medicine, the significant disparity in the inclusion of women in the guidelines’ writing groups cannot fully explain the paucity of data on GM [7,38]. Differences in referrals to sex differences in guidelines involving more or less than 30% of female authors and in those with relevant female authorship vs. male authorship are mostly not significant. Furthermore, the qualification of the members of the writing groups, both males and females, and the accurate methodology applied in generating the guidelines should reassure readers regarding the avoidance of major biases in the collection and interpretation of published evidence. Corroborating this hypothesis, our research reveals a low number of scientific works cited in the bibliography with titles concerning sex and gender differences. Although we cannot exclude the existence of other cited scientific works reporting sex and gender differences in the text without mentioning it in the title, the number of citations identified is very low (with the exception of two guidelines, it is less than 3%). Therefore, the problem appears not to be in the guidelines but in a lack of attention to sex and gender differences in the scientific articles at the basis of guideline writing. In fact, despite well-established sex differences, studies comparing cardiovascular outcomes in females are lacking at both the preclinical and clinical levels. Furthermore, there are no specific recommendations or guidelines for treating cardiovascular disease in females compared to males, including cardiovascular therapies with recognized sex differences such as those targeting the renin–angiotensin system [39].

Nonetheless, female representation in guidelines’ authorship should increase, reflecting ESC Gender Policy, developed during the leadership of Prof. Stephan Achenbach in 2020–2022. In our opinion, all guidelines should acknowledge that the available evidence is insufficient to give gender-specific recommendations [40]. However, among the 23 guidelines examined, only 6 [18,22,24,26,29,30] reported this limitation in the “gaps of knowledge” section.

In a previous paper, we analyzed the attention paid to gender in major trials on hypertension treatment and found that it was insufficient [41]. The implementation of the rules proposed for balanced gender representation in the design and analysis of scientific works could be useful to improve the lack of evidence in the field of GM [42]. It appears mandatory to implement current and future clinical trials to account for sex and gender differences not only in epidemiology but also in diagnostic and therapeutic pathways. Factors associated with diagnostic inertia may vary between sexes, as suggested by a study based on the Spanish ESCARVAL-RISK cohort [43]. Regarding the therapeutic perspective, identifying sex-specific targets may help guide therapeutic choices and improve therapeutic efficacy. Another key point in addressing sex-associated differences in therapeutics involves prescription biases. Previous studies have shown how low-value practices leading to adverse drug events were more frequent in women compared to men [44].

The present study has important limitations worth mentioning. References on GM were screened based on the title and not the content of the cited paper. Thus, we cannot guarantee that all references on GM have been detected. Additionally, the timeframe of the guidelines’ selection may have led to us underestimating the improvements in the attention paid to GM in recent guidelines compared to older ones. However, considering the same number of years before and after the development of the ESC Gender Policy is of crucial importance to understand its impact and to provide an accurate picture of the current situation.

## Figures and Tables

**Table 1:** Guidelines produced by the European Society of Cardiology (ESC) from 2018 to 2023.

N°	Title	Year	Pages	References
1	ESC Guidelines for the management of cardiovascular diseases during pregnancy [8]	2018	84	439
2	ESC/EACTS Guidelines on myocardial Revascularization [9]	2018	96	786
3	ESC Guidelines for the diagnosis and management of syncope [10]	2018	69	440
4	Fourth universal definition of myocardial infarction [11]	2018	33	201
5	ESC/ESH Guidelines for the management of arterial hypertension [12]	2018	98	629
6	ESC Guidelines for the diagnosis and management of acute pulmonary embolism developed in collaboration with the European Respiratory Society (ERS) [13]	2019	61	478
7	ESC/EAS Guidelines for the management of dyslipidaemias: lipid modification to reduce cardiovascular risk [14]	2019	78	608
8	ESC Guidelines for the diagnosis and management of chronic coronary syndromes [15]	2019	71	529
9	ESC Guidelines for the management of patients with supraventricular tachycardia [16]	2019	66	598
10	ESC Guidelines for the management of adult congenital heart disease [17]	2020	84	329
11	ESC Guidelines for the diagnosis and management of atrial fibrillation developed in collaboration with the European Association for Cardio-Thoracic Surgery (EACTS) [18]	2020	126	1491
12	ESC Guidelines on sports cardiology and exercise in patients with cardiovascular disease [19]	2020	80	623
13	ESC Guidelines on cardiovascular disease prevention in clinical practice [20]	2021	111	837
14	ESC Guidelines for the diagnosis and treatment of acute and chronic heart failure [21]	2021	128	1001
15	ESC Guidelines on cardiac pacing and cardiac resynchronization therapy [22]	2021	94	844
16	ESC/EACTS Guidelines for the management of valvular heart disease [23]	2022	72	562
17	ESC/ERS Guidelines for the diagnosis and treatment of pulmonary hypertension [24]	2022	114	855
18	ESC Guidelines on cardiovascular assessment and management of patients undergoing non-cardiac surgery [25]	2022	99	705
19	ESC Guidelines for the management of patients with ventricular arrhythmias and the prevention of sudden cardiac death [26]	2022	130	1155
20	ESC Guidelines on cardio-oncology developed in collaboration with the European Hematology Association (EHA), the European Society for Therapeutic Radiology and Oncology (ESTRO) and the International Cardio-Oncology Society (IC-OS) [27]	2022	133	837
21	ESC Guidelines for the management of acute coronary syndromes [28]	2023	107	936
22	ESC Guidelines for the management of endocarditis [29]	2023	95	857
23	ESC Guidelines for the management of cardiovascular disease in patients with diabetes [30]	2023	98	845
24	ESC Guidelines for the management of cardiomyopathies [31]	2023	124	1204

**Table 2:** Summary of female representation among authors of the guidelines and the type of sections dedicated to gender-specific medicine (GM).

Guideline N°	Number of Women among Authors	Sections Dedicated to Sex Differences	Sections Dedicated to Pregnancy	Mentions to GM in “Key Points”	Mentions to GM in “Gaps in Knowledge”
1	10/20 (50%) First and Last Author	NA	NA	NA	NA
2	1/22 (4.5%)	NO	NO	NO	NO
3	3/16 (18.7%)	NO	NO	NO	NO
4	0/7 (0%)	NO	NO	NO	NO
5	3/28 (10.7%)	NO	B	NO	NO
6	6/23 (26%)	NO	A	NO	NO
7	7/21 (33.3%)	NO	C	NO	NO
8	3/25 (12%)	B	NO	NO	NO
9	3/21 (14.3%)	NO	A	YES	NO
10	7/17 (41.2%) Last Author	C	C	YES	NO
11	7/24 (29.2%) Last Author	A	B	NO	YES
12	5/24 (20.8%)	A	NO	NO	NO
13	9/30 (30%)	C	D	NO	NO
14	10/31 (32.2%) First and Last Author	NO	B	NO	NO
15	2/24 (8.3%)	B	B	NO	NO
16	3/21 (14.2%)	NO	B	NO	YES
17	7/29 (24.1%)	NO	D	NO	NO
18	8/29 (27.6%) First Author	A	NO	NO	YES
19	6/25 (24%) First Author	NO	NO	NO	YES
20	10/30 (33.3%) Last Author	NO	B	NO	NO
21	8/26 (30.8%)	A	C	NO	NO
22	9/25 (36%) First Author	A	B	NO	YES
23	8/22 (36.4%)	C	C	NO	NO
24	9/27 (33.3%) First Author	A	C	NO	YES
**TOT**	**144/567 (25.4%)** **First or Last Authorship 9/24 (37.5%)**	**11 (47.8%)** **(6 A; 2 B; 3 C)**	**16 (69.6%)** **(2 A; 7 B; 5 C; 2 D)**	**2 (8.7%) YES**	**6 (26.1%) YES**

Title and reference of each specific guideline number (Guideline N°) are reported in Table 1. In the last row, cumulative results of all guidelines are reported. GM stands for gender-specific medicine. NA = not applicable; A = chapter; B = section; C = sub-section; D = sub-sub-section.

**Table 3:** Number of times sex or gender differences were mentioned in the context of epidemiology, diagnosis, and therapy, and number of citations referring to sex or gender in the bibliography of each guideline.

Guideline N°	Mentions of Sex Differences in Epidemiology	Mentions of Sex Differences in Diagnosis	Mentions of Sex Differences in Treatment	N° of Citations (%) Referring to Sex or Gender
1	NA	NA	NA	NA
2	1	1	0	0 (0%)
3	2	1	0	7 (1.6%)
4	4	3	0	6 (1.4%)
5	16	3	7	7 (3.5%)
6	3	4	0	5 (1.0%)
7	4	6	2	13 (2.1%)
8	2	5	2	29 (5.5%)
9	4	0	0	4 (0.7%)
10	4	2	9	8 (2.4%)
11	11	1	6	34 (2.3%)
12	13	5	2	10 (1.6%)
13	15	3	1	54 (1.5%)
14	6	1	2	13 (1.3%)
15	3	0	3	7 (0.8%)
16	4	2	1	8 (1.4%)
17	8	1	0	2 (0.2%)
18	2	3	1	7 (1.0%)
19	10	1	1	7 (0.6%)
20	1	4	0	21 (2.5%)
21	2	1	0	24 (2.6%)
22	5	3	1	5 (0.6%)
23	4	2	2	18 (2.1%)
24	15	6	1	27 (2.2%)
**Median**	**4 (IQR 8)**	**2 (IQR 3)**	**1 (IQR 2)**	**8 (IQR 15), 1.5% (IQR 1.5)**

Title and reference of each specific guideline number (Guideline N°) are reported in Table 1. In the last row, median and interquartile ranges (IQR) of the number of mentions of gender-specific medicine (GM) are reported. NA stands for not applicable.

## Data Availability

All data are available and reported in the tables of the manuscript.
